# Nuclear Syndecan-1 Regulates Epithelial-Mesenchymal Plasticity in Tumor Cells

**DOI:** 10.3390/biology10060521

**Published:** 2021-06-11

**Authors:** Ashish Kumar-Singh, Malgorzata Maria Parniewska, Nikolina Giotopoulou, Joman Javadi, Wenwen Sun, Tünde Szatmári, Katalin Dobra, Anders Hjerpe, Jonas Fuxe

**Affiliations:** 1 Department of Laboratory Medicine, Karolinska Institutet, Division of Pathology, SE-14186 Stockholm, Sweden; ashish.kumar.singh@ki.se (A.K.-S.); malgorzata.parniewska@ki.se (M.M.P.); nikolina.giotopoulou@ki.se (N.G.); joman.javadi@ki.se (J.J.); wenwen.sun@ki.se (W.S.); tunde.szatmari@ki.se (T.S.); anders.hjerpe@ki.se (A.H.); 2 Division of Clinical Pathology/Cytology, Karolinska University Laboratory, Karolinska University Hospital, SE-14186 Stockholm, Sweden

**Keywords:** syndecan-1, SDC1, nuclear translocation, epithelial-mesenchymal transition, plasticity, TGF-β1

## Abstract

**Simple Summary:**

The major cause of death in cancer is that tumor cells metastasize (i.e., spread to vital organs such as the lungs and liver). Our knowledge of how metastasis occurs is incomplete and consequently, there is a lack of therapeutic strategies that target metastasis. Recent data show that inflammatory signals may influence tumor cells to undergo an identity switch, whereby they develop metastatic properties. Understanding how this identity switch is regulated is important to develop novel therapeutics. Recently, syndecan-1, a protein found on the surface of various cells, was shown to regulate the identity switch. In this study, we report that re-localization of syndecan-1 from the cell surface to the nucleus contributes to its capacity to regulate the identity switch in tumor cells. These results identify nuclear syndecan-1 as a regulator of the identity switch and open up to further studies to elucidate the mechanisms involved.

**Abstract:**

Tumor cells undergoing epithelial-mesenchymal transition (EMT) lose cell surface adhesion molecules and gain invasive and metastatic properties. EMT is a plastic process and tumor cells may shift between different epithelial-mesenchymal states during metastasis. However, how this is regulated is not fully understood. Syndecan-1 (SDC1) is the major cell surface proteoglycan in epithelial cells and has been shown to regulate carcinoma progression and EMT. Recently, it was discovered that SDC1 translocates into the cell nucleus in certain tumor cells. Nuclear SDC1 inhibits cell proliferation, but whether nuclear SDC1 contributes to the regulation of EMT is not clear. Here, we report that loss of nuclear SDC1 is associated with cellular elongation and an E-cadherin-to-N-cadherin switch during TGF-β1-induced EMT in human A549 lung adenocarcinoma cells. Further studies showed that nuclear translocation of SDC1 contributed to the repression of mesenchymal and invasive properties of human B6FS fibrosarcoma cells. The results demonstrate that nuclear translocation contributes to the capacity of SDC1 to regulate epithelial-mesenchymal plasticity in human tumor cells and opens up to mechanistic studies to elucidate the mechanisms involved.

## 1. Introduction

The initial steps of the metastatic process involve induction of epithelial-to-mesenchymal transition (EMT), whereby tumor cells shift identity from a stationary, epithelial phenotype to a more motile, mesenchymal-like phenotype. EMT is not a binary switch but rather a dynamic process by which tumor cells adopt various states of epithelial/mesenchymal phenotypes. This plasticity is a prerequisite for changes in tumor cell morphology during invasion, intravasation, and extravasation along the metastatic route [1]. The mechanisms involved in regulating epithelial-to-mesenchymal plasticity in tumor cells are not fully understood.

The induction of EMT involves transcriptional re-programming, which is driven by a set of core EMT transcription factors (EMT-TFs) belonging to the Snail, Twist, and Zeb families [2,3,4]. Cytokine transforming growth factor-β1 (TGF-β1), which is overexpressed in many cancers, is a potent inducer of EMT [5]. Induction of an EMT program in response to TGF-β1 involves cooperation between Smads and core EMT-TFs [6,7]. EMT is associated with a change in the repertoire of adhesion molecules that are expressed and localized at the cell surface. One example of this is the “cadherin switch”, which refers to downregulation of E-cadherin and concomitant upregulation of N-cadherin. The cadherin switch is a hallmark of EMT and is associated with malignant progression of epithelial cancers into invasive and metastatic disease [8]. Another example is the coxsackie- and adenovirus receptor (CXADR/CAR), a tight junction-based cell adhesion molecule, which is frequently lost during carcinoma progression. Recently, we found that loss of CXADR leads to hyperactivation of the AKT signaling pathway and sensitizes breast cancer cells to TGF-β1-induced EMT [9]. Thus, some cell surface molecules are important to maintain epithelial characteristics and control cellular sensitivity to TGF-β1-induced EMT.

Syndecan-1 (SDC1) is the major cell surface heparan sulfate proteoglycan in epithelial cells [10]. SDC1 interacts with various components of the extracellular matrix and has been found to bind various growth factors and to stabilize their interaction with receptors, which influences the activity of different signaling pathways. Loss of SDC1 is detected during dedifferentiation and the progression of various epithelial cancers and is associated with EMT [11]. Simultaneous loss of SDC1 and E-cadherin was also reported in embryonic EMT [12]. Experimentally, it has been shown that silencing of SDC1 in epithelial cells results in decreased expression of E-cadherin, and the acquisition of a more invasive, mesenchymal phenotype [13]. Conversely, overexpression of SDC1 was found to promote an epithelial morphology and inhibit cell proliferation in mammary epithelial cells [14] and to suppress EMT in oral cancer cells [15]. Changes in the expression of SDC1 has also been linked to EMT markers in colorectal cancer [16]. Moreover, the core EMT-TF ZEB1 was identified as a transcriptional repressor of SDC1 in prostate cancer cells [17]. Together, these results indicate that SDC1 promotes epithelial integrity and is involved in the EMT process.

Although a majority of studies have examined the role of SDC1 at the cell surface, it has been discovered that SDC1 can translocate to the nucleus in different cells [18]. Nuclear translocation of SDC1 has been linked to reduced proliferation of mesothelioma cells and occurs in a tubulin-dependent manner [18,19]. The translocation is dependent on the presence of an RMKKK motif in the cytoplasmic tail, which functions as a nuclear localization signal (NLS) [20]. Our previous studies have shown that nuclear translocation of SDC1 is delayed after exposure to TGF-β [19,21]. Moreover, it has been found that the amount of nuclear SDC1 is reduced after expression of the enzyme heparanase, which is upregulated in many cancers and promotes tumor progression and metastasis [22]. However, whether nuclear SDC1 regulates the EMT process and tumor cell invasion is not known. The aim of the present study was therefore to test the hypothesis that nuclear SDC1 is involved in regulating epithelial-mesenchymal plasticity in tumor cells.

## 2. Materials and Methods

### 2.1. Cell Culture

B6FS human fibrosarcoma cells [23] were grown in RPMI 1640 + glutaMAX^TM^-I (Gibco/Thermo Fisher Scientific, Gothenburg, Sweden) supplemented with 10% fetal bovine serum (FBS) and Gentamicin 1 mg/mL (Gibco). All cells were cultured in 75 cm^2^ tissue culture flasks (Sarstedt, Newton, NC, USA) in humidified 5% (*v*/*v*) CO_2_ at 37 °C and the culture medium was changed twice a week. The human lung adenocarcinoma A549 cell line was obtained from ATCC (Teddington, UK). A549 cells were maintained in medium (DMEM) + glutaMAX^TM^-I (Gibco) supplemented with 10% FBS and 1% penicillin/streptomycin (all from Thermo Fisher Scientific) in a humidified atmosphere of 5% CO_2_ at 37 °C. Recombinant human TGF-β1 was purchased from R&D Systems (Minneapolis, MN, USA). Human prostate cancer PC3 cells were cultured in DMEM supplemented with 10% FBS and 1% penicillin/streptomycin (Thermo Fisher Scientific) in a humidified atmosphere of 5% CO_2_ at 37 °C.

### 2.2. Fluorescence Activated Cell Sorting (FACS)

B6FS fibrosarcoma cells were stably transfected with cDNA vectors expressing either full length SDC1 (FL), a mutant variant of SDC1 lacking the RMKKK sequence (RMKKKdel) or an empty vector (EV) as previously described [24]. Cell surface expression of SDC1 was determined by FACS analysis prior to the experiments. Cells were detached with enzyme-free cell dissociation buffer (13151-014, Thermo Fisher Scientific) for 15 min. Following fixation in 2% buffered formaldehyde for 10 min at 37 °C, cells were incubated with antibodies against CD138 (1:20 dilution; MCA2459, Bio-Rad Laboratories, Solna, Sweden) for 15 min at 4 °C and Alexa 488-conjugated goat anti-mouse secondary antibody (A-110017; Invitrogen/ Thermo Fisher Scientific) for 15 min at room temperature.

### 2.3. RNA Interference

Two hundred thousand A549 cells/well were seeded and after one day transfected using Lipofectamine RNAIMAX (Thermo Fisher Scientific). A pool of three different siRNA constructs specific for SDC1 were used (Ambion/Thermo Fisher Scientific) with an optimized concentration of 10 µM. Scrambled siRNA with no target mRNA was used as the negative control. SDC1 specific siRNA or scrambled control siRNA and lipofectamine were diluted in antibiotics- and serum-free medium according to the manufacturer’s instructions and incubated for 5 min. The different mediums containing SDC1 specific or scrambled control siRNA-lipofectamine complexes were then added separately to the cells and incubated for 24 h at 37 °C and 5% CO_2_.

### 2.4. Protein Extraction and Immunoblot Analysis

Cells were washed with ice-cold PBS and lysed with RIPA buffer (Thermo Fisher Scientific) containing Halt Protease and Phosphatase Inhibitor Cocktail (Thermo Fisher Scientific). Protein concentrations were determined with the BCA assay (Bio-Rad) using bovine serum albumin as the standard. For immunoblot analysis, 10–30 µg protein was loaded to any kD mini- protean TGX Gels (Bio-Rad) and transferred to PVDF membranes using the trans-blot turbo transfer system (Bio-Rad). Membranes were blocked at room temperature for 40 min in 3% nonfat dry milk in TBS-T and incubated with primary antibodies over night at 4 °C. The following antibodies were used: rabbit polyclonal antibody against E-cadherin (1:1000, Cell Signaling, Leiden, The Netherlands), vimentin (1:1000, Cell Signaling), N-cadherin (1:1000, Cell Signaling), GAPDH (1:1000, Cell Signaling), syndecan-1 (1:1000, Abcam, Cambridge, UK). Following washing, the membrane was incubated with secondary antibodies (Donkey Anti-Rabbit or Sheep Anti-Mouse IgG, F (ab’)2 Fragment Specific, Peroxidase Conjugated (Thermo Fisher Scientific) for 1 h at room temperature. For chemiluminescent detection, chemiluminescent HRP Substrate (K-12043-D10; Advansta/AH Diagnostics, Solna, Sweden) was added and the membrane was incubated for 1 min. The Odyssey Imaging System (LI-COR Biotechnology, Bad Homburg, Germany) was used to detect chemiluminescence and the relative expression was normalized to GAPDH as the loading control using ImageJ software (https://imagej.nih.gov/ij/).

### 2.5. Quantitative Real-Time RT–PCR Analysis

Total RNA was extracted using an RNeasy Mini Kit (Qiagen, Valencia, CA, USA) according to the manufacturer’s instructions. cDNA synthesis was performed by using the QuantiTect Reverse Transcription Kit (Qiagen, Valencia, CA, USA) using an amount of 1 μg of total RNA. For qPCR (quantitative real time PCR) analysis, 5 ng of the cDNA mixture was used for PCR amplification by a QuantiTect SYBR Green PCR Kit (Qiagen, Valencia, CA, USA) with validated QuantiTect primers (Qiagen, Valencia, CA, USA). The following genes were analyzed: SDC1 (QT00037128), vimentin (QT00095795), and GAPDH (QT00079247). The PCR was carried out as follows: 3 min at 95 °C followed by 35 cycles of 3 s at 95 °C, 20 s at 55 °C and 2 s extension step at 72 °C in a BioRad PCR system.

### 2.6. Immunofluorescence Staining and Quantitative Analysis

Following transfection, 15,000 cells were seeded to 24 well plates with cover slips. After 24 h, cells were treated with 10 ng/mL TGF-β1 (240-B-002; R&D Systems, Abingdon, UK) for 48 or 72 h.

Cells were fixed with 4% PFA for 15 min and permeabilized with 0.1% PBST for 15 min. Blocking was performed in 5% donkey serum + 2% BSA in 0.1% PBST. Primary antibody staining was performed at 4 °C overnight with the following concentrations: anti-syndecan1 (Abcam, ab128936, rabbit) 1:500, anti-vimentin (HPA027524, Sigma-Aldrich, Stockholm Sweden, rabbit) 1:50, anti-ZEB1 (HPA027524, Sigma-Aldrich, rabbit) 1:50. Secondary antibody staining using Alexa Fluor 488 goat anti-rabbit (A32731, Thermo Fisher Scientific) or Alexa Fluor 555 goat anti-rabbit (A21428, Thermo Fisher Scientific) was performed for 1 h at room temperature. Antibodies were diluted in 0.1% BSA in 0.1% PBST. Cover slips were mounted using Vectashield Antifade Mounting Medium with DAPI (H-1200-10, Vector Laboratories). Images were captured using a Zeiss Observer Z1 inverted microscope and a Zeiss LSM800 confocal microscope.

Nuclear SDC1 in A549 cells was quantified from Z-stack images acquired with a Zeiss LSM800 confocal microscope using a 100× objective (2–3 cells per field of view). Images were analyzed using ImageJ/Fiji. Z-stack slices containing nuclei were isolated and Sum Intensity Projection was generated. Nuclei were segmented using the DAPI signal and the mean intensity of SDC1 was quantified within the segmented region. On average, a total of 15 cells from three wells were analyzed. Membrane SDC1 in A549 cells was quantified from images obtained from a Zeiss Observer Z1 inverted microscope using 40× objective. Acquired images were analyzed using CellProfiler [25]. Nuclei were segmented based on DAPI staining. Membrane SDC1 1 was identified by measuring SDC1 signal intensity outside the nuclei. A total of 5-field images per well (average of 15 cells per field of view) and a total of three wells were analyzed. Data were represented as mean values per well.

ZEB1 in A549 cells was quantified from images obtained from a Zeiss Observer Z1 inverted microscope using 40× objective. Acquired images were analyzed using CellProfiler [25]. Nuclei were segmented based on DAPI staining. ZEB1 intensity was quantified within the segmented region. A total of 5-field images per well (average of 15 cells per field of view) and a total of three wells were analyzed. Data were represented as mean values per well.

Vimentin in A549 cells was quantified from images obtained from a Zeiss LSM800 confocal microscope using a 40× objective. Acquired images were analyzed using CellProfiler [25]. Nuclei were segmented based on DAPI staining. Vimentin was identified by measuring vimentin signal intensity outside the nuclei. Area density of vimentin stain per cell was measured. A total of 7-field images per well (average of 15 cells per field of view) were acquired. Data were represented as mean vimentin area per well.

Nuclear SDC1 in BSF6 cell lines was quantified from Z-stack images acquired with a confocal microscope (100×, field of view including an average of 15 cells). Images were analyzed using ImageJ/Fiji. Nuclei were segmented using the DAPI signal on a single focal plane corresponding to the cross-section of all nuclei. Mean intensity of SDC1 was quantified withing the segmented regions. On average, 45 cells from three wells were analyzed.

ZEB1 in BSF6 cell lines was quantified from images obtained from a Zeiss Observer Z1 inverted microscope using a 40× objective. Acquired images were analyzed using CellProfiler [25]. Nuclei were segmented based on DAPI staining. ZEB1 intensity was quantified within the segmented region. A total of 4-field images per well (average of 15 cells per field of view) and a total of two wells were analyzed. Data were represented as mean values per well.

### 2.7. Invasion Assays

Invasion assays were performed by using 8-μm pore cell culture inserts (Merck Chemicals and Life Science, Stockholm, Sweden) and 3 mm cylinders. B6FS cells (stably overexpressing FL SDC1, the RMKKKdel mutant or EV) were trypsinized and quantified. A number of 50,000 cells were seeded on top of growth factor-reduced Matrigel (3 mg/mL, AH Diagnostics) diluted 1:10 in DMEM medium into the cell culture inserts. The cell culture inserts were placed in 24-well plates in which 700 μL of medium, either control or + TGF-β1 10 ng/mL, had been added. After 4 h, in order to study the number of cells that had migrated in the lower chamber of the 24 wells, the cells were counted using Alamar blue and Countess (Invitrogen/Thermo Fisher Scientific) for three separate measurements in each of the triplicates for each sample. A similar procedure was used for A549 cells transfected with Scr or SDC siRNA and either left untreated or treated with TGF-β1 (10 ng/mL) for 30 h. A total of 30,000 A549 cells were added on top of the Matrigel.

### 2.8. Gene Expression Analysis

Analysis of SDC1 mRNA expression in human cancer cells was performed using the Broad Institute Cancer Cell Line Encyclopedia (https://portals.broadinstitute.org/ccle (accessed on 15 March 2021)).

### 2.9. Statistics

Statistical analyses were performed using GraphPad Prism 9 software. Analysis of variance (ANOVA) followed by Tukey post-hoc tests were used to compare means and statistical differences between groups. Significant differences are indicated by: * (*p* < 0.05), ** (*p* < 0.01), *** (*p* < 0.001), **** (*p* < 0.0001).

## 3. Results

### 3.1. Loss of Nuclear SDC1 during TGF-β1-Induced EMT in A549 Cells

**Figure 1:**
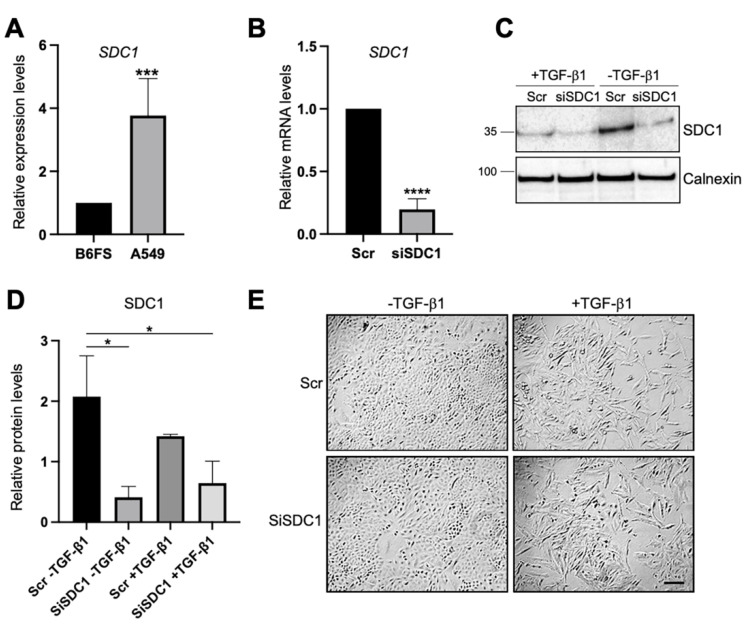
Knockdown of SDC1 during TGF-β1-induced EMT in A549 cells. (**A**) Bar graph showing relative *SDC1* mRNA expression in A549 cells compared to B6FS cells. Values were normalized to GAPDH and B6FS cells. (**B**) qPCR results showing *SDC1* mRNA levels in A549 cells transfected with SDC1 siRNA (siSDC1) compared to scrambled siRNA (Scr). (**C**) Western blot results showing the effect of SDC1 knockdown and TGF-β1 treatment (10 ng/mL, 72 h) on SDC1 protein levels. Calnexin was used as a loading control. (**D**) Bar graph showing quantification of western blot results. (**E**) Brightfield images showing morphological changes characteristic of EMT in A549 cells transfected with Scr or SDC1 siRNA and treated with TGF-β1 (10 ng/mL, 48 h). Scale bar = 20μm. * = *p* < 0.05; *** = *p* < 0.001; **** = *p* < 0.0001.

Loss- and gain-of-function studies were used to elucidate the possible role of nuclear SDC1 in regulating EMT in two human tumor cell models: (i) human lung A549 adenocarcinoma cells, which are of epithelial origin but have the capacity to undergo EMT after exposure to TGF-β1 [26], and (ii) human B6FS fibrosarcoma cells, which are of mesenchymal origin and have been used in previous studies to identify a role of nuclear SDC1 in regulating cell proliferation [27]. qPCR analysis showed that *SDC1* mRNA expression was almost 4-fold higher in A549 cells compared to B6FS cells (Figure 1A), which was in line with RNA sequencing data from the Broad Institute Cancer Cell Line Encyclopedia (https://portals.broadinstitute.org/ccle (accessed on 15 March 2021)), showing higher *SDC1* mRNA levels in tumor cells with epithelial origin including lung cancer cells compared to tumor cells with mesenchymal origin (Supplementary Figure S1). Knockdown of SDC1 in A549 cells was performed by siRNA and the effect was confirmed by qPCR (Figure 1B). No signs of toxic side effects were observed in transfected cells (Supplementary Figure S2A). Further analysis by western blot showed that cells transfected with SDC1 siRNA expressed lower levels of SDC1 also during TGF-β1-induced EMT (Figure 1C,D). A549 cells transfected with either Scr or SDC1 siRNA acquired an elongated morphology upon the induction of EMT by exposure to recombinant TGF-β1 (10 ng/mL, 72 h) (Figure 1E).

**Figure 2:**
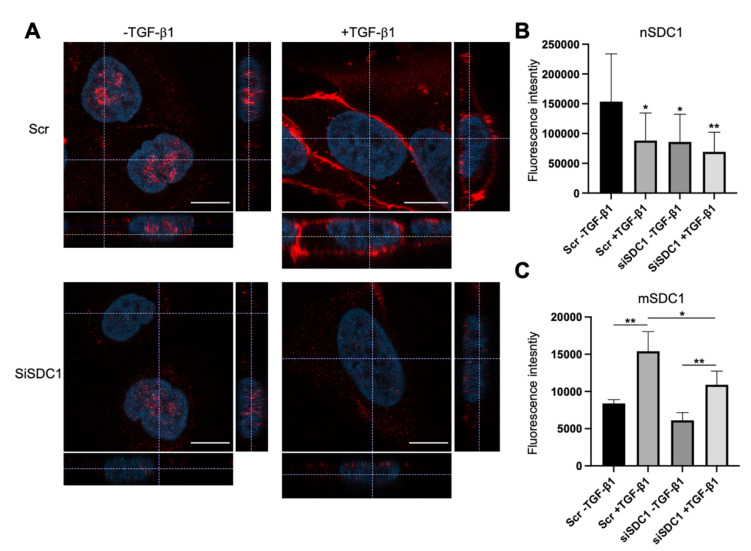
Loss of nuclear SDC1 during TGF-β1-induced EMT in A549 cells. (**A**) Confocal immunofluorescent images showing staining and localization of SDC1 (red) in A549 cells transfected with Scr or SDC1 siRNA and either left untreated or treated with TGF-β1 (10 ng/mL, 48 h). Cell nuclei were stained with DAPI (blue). Scale bars = 3 μm. (**B**,**C**) Bar graphs showing fluorescence intensity of nuclear SDC1 staining (nSDC1, B) and membrane SDC1 staining (mSDC1, C) (**D**). * = *p* < 0.05; ** = *p* < 0.01.

Immunofluorescence staining and confocal microscopy analysis was performed to elucidate to what extent SDC1 was localized to the nucleus in A549 cells and whether this was affected by SDC1 knockdown and TGF-β1 treatment. Positive nuclear staining and a weaker membrane staining of SDC1 was detected in the control A549 cells (Figure 2A). In contrast, nuclear SDC1 was weaker while membrane SDC1 was stronger after TGF-β1-induced EMT (Figure 2A–C and Supplementary Figure S2B). Loss of nuclear SDC1, but not membrane SDC1, was detected in siSDC1-transfected A549 cells and a similar shift in SDC1 localization from the nucleus to the membrane was seen in these cells upon TGF-β1 exposure. Together, these results demonstrated that nuclear SDC1 was lost and translocated to the plasma membrane during TGF-β1-induced EMT.

### 3.2. Loss of SDC1 Promotes EMT in A549 Cells

**Figure 3:**
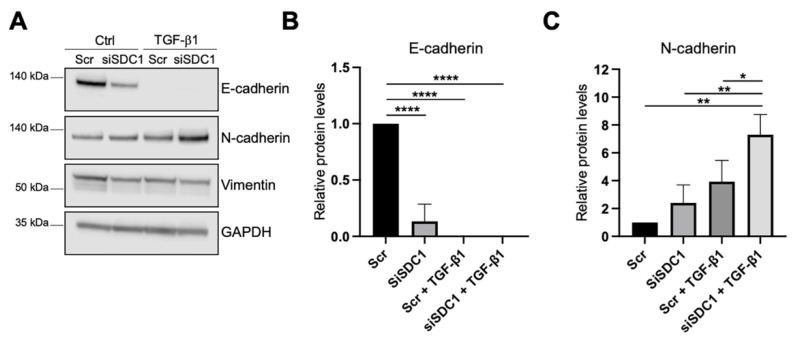
Effect of SDC1 knockdown on TGF-β1-induced EMT in A549 cells. (**A**) Western blot results showing the effect of SDC1 knockdown and TGF-β1 treatment (10 ng/mL, 48 h) on E-cadherin, vimentin, and N-cadherin protein levels in A549 cells. GAPDH was used as a loading control. (**B**,**C**) Bar graphs showing quantification of western blot results from studying the effect of SDC1 knockdown and TGF-β1 treatment (10 ng/mL, 72 h) on protein levels of E-cadherin (**B**) and N-cadherin (**C**). Values were normalized to GAPDH and related to the levels of each protein in untreated Scr control cells. The results represent mean values of three independent experiments. * = *p* < 0.05; ** = *p* < 0.01; **** = *p* < 0.0001.

As a next step, we analyzed whether knockdown of SDC1 affected the EMT response in A549 cells. As one of the hallmarks of EMT is an E-to-N-cadherin switch, we studied if knockdown of SDC1 affected these markers. E-cadherin levels were significantly reduced in SDC1 knockdown cells compared to the control cells, and were not detectable after TGF-β1 treatment (10 ng/mL, 72 h) (Figure 3A,B). N-cadherin levels were not affected by SDC1 knockdown alone but were significantly more induced in SDC1 knockdown cells compared to the control cells after TGF-β1 treatment (Figure 3A,C). In comparison, vimentin levels were not affected by knockdown of SDC1, neither under baseline conditions nor after TGF-β1 treatment (Figure 3A and Supplementary Figure S2C). Immunofluorescence staining showed loss of E-cadherin upon knockdown of SDC1 but no significant effect on CXADR (Supplementary Figure S2B). However, E-cadherin and CXADR were significantly reduced after TGF-β1 treatment (10 ng/mL, 72 h), both in the control and siSDC1 cells.

**Figure 4:**
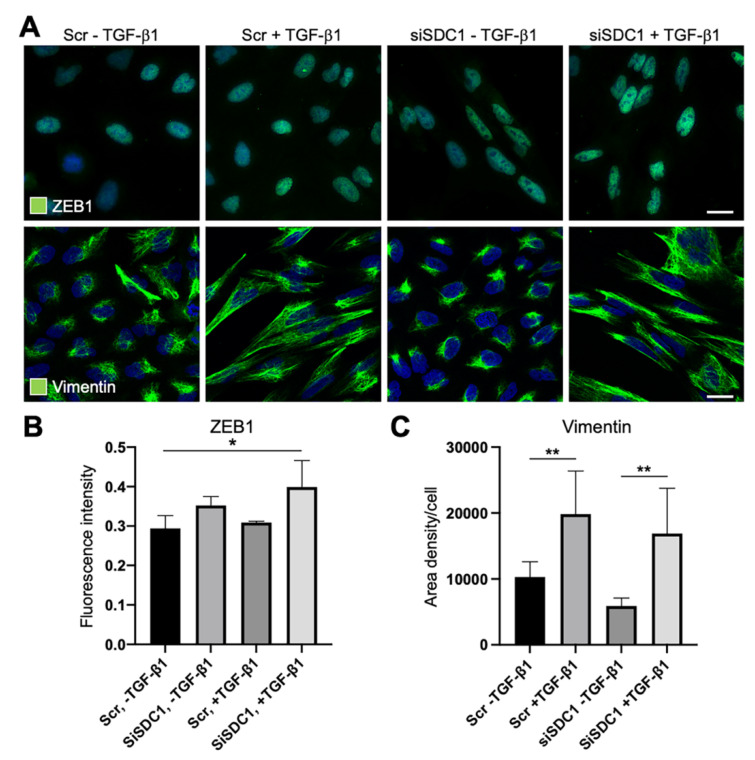
Immunofluorescence analysis of the effect of SDC1 knockdown on TGF-β1-induced EMT in A549 cells. (**A**) Immunofluorescence images showing staining of ZEB1 and vimentin in A549 cells transfected with scrambled control siRNA (Scr) or SDC1 siRNA (siSDC1), and thereafter left untreated or treated with TGF-β1 (10 ng/mL, 48 h). Cell nuclei were counterstained with DAPI (blue). Scale bars = 5 μm. (**B**,**C**) Bar graphs showing results from image-based analysis and quantification of the effect of SDC1 knockdown and TGF-β1 exposure on the intensity of ZEB1 staining (**B**) and area density of vimentin (**C**). * = *p* < 0.05; ** = *p* < 0.01.

Considering reports indicating that deregulation of SDC1 during EMT is linked to the core EMT-TF ZEB1, we studied whether ZEB1 was affected by SDC1 knockdown. Immunofluorescence staining showed weak nuclear staining of ZEB1 in untreated control cells and SDC1 knockdown cells (Figure 4A,B). Upon TGF-β1 exposure, nuclear staining of ZEB1 increased in SDC1 knockdown cells compared to control cells. These data suggested that knockdown of SDC1 enhanced the EMT response downstream of TGF-β1. To analyze whether knockdown of SDC1 affected morphological changes induced during EMT, we performed immunostaining for vimentin, an intermediate filament protein regulating cell shape. The cellular elongation, which was induced during TGF-β1-induced EMT, was clearly associated with a more widespread distribution of vimentin (Figure 4A,C). However, no difference was found between the control cells and SDC1 knockdown cells. In line with this, we found that A549 cells acquired a more elongated nuclear shape after TGF-β1 exposure, with no difference between SDC1 knockdown cells and control cells (Supplementary Figure S2D).

### 3.3. Nuclear SDC1 Reduces Mesenchymal Properties of B6FS Fibrosarcoma Cells

The results from the A549 cells indicated that loss of nuclear SDC1 is linked to TGF-β1-induced epithelial-mesenchymal plasticity. As a second approach to study this, we analyzed whether nuclear translocation of SDC1 would affect EMT characteristics in mesenchymal tumor cells. For these studies, we used human B6FS fibrosarcoma cells that had previously been stably transfected with plasmid constructs expressing either full-length (FL) SDC1, a mutated variant of SDC1 carrying a deletion of the nuclear localization signal RMKKK (RMKKKdel), or an empty vector (EV) [28].

**Figure 5:**
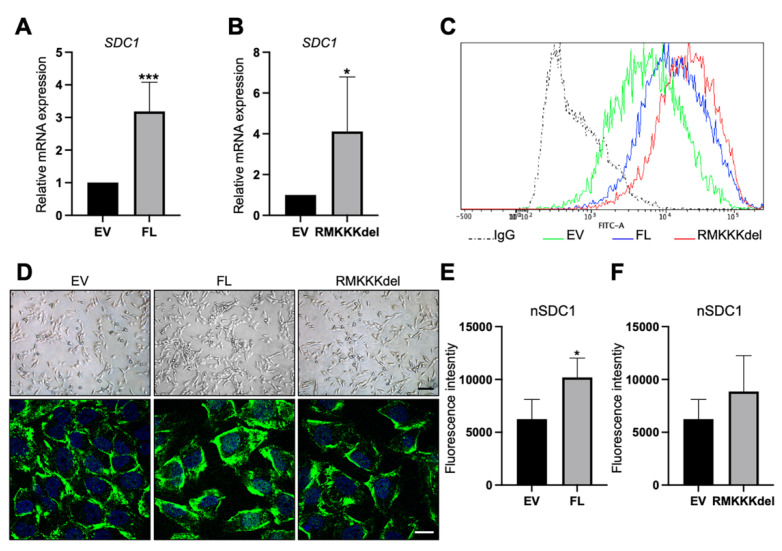
Overexpression of SDC1 in B6FS fibrosarcoma cells. (**A**,**B**) Bar graphs showing results from qPCR analysis of *SDC1* mRNA levels in B6FS cells transfected with full length SDC1 (FL, **A**) or a mutant SDC1 lacking the nuclear localization signal RMKKK (RMKKKdel, **B**) compared to B6FS cells transfected with an empty vector (EV). (**C**) Histogram showing cell surface levels of SDC1 in B6FS cells as detected by fluorescence activated cell sorting (FACS) analysis. Dotted line represents the results obtained with an unspecific IgG antibody, which was used as a negative control. Solid lines show the cell surface levels of SDC1 in cells stably overexpressing the empty vector (EV, green), FL SDC1 (FL, blue), or the SDC1 mutant (RMKKKdel, red). (**D**) Upper panels: Brightfield images showing the morphology of B6FS cells transfected with the empty vector (EV), FL SDC1, or the RMKKKdel mutant. Lower panels: Images showing immunofluorescent staining of SDC1 (green) in B6FS EV, FL, and RMKKKdel cells. Scale bars = 30 μm (upper); 5 μm (lower). (**E**,**F**) Bar graphs showing quantification of nuclear SDC1 in confocal microscopy images of B6FS cells transfected with FL SDC1 (**E**) or the RMKKKdel mutant (**F**) compared to the control cells. * = *p* < 0.05; *** = *p* < 0.001.

B6FS cells transfected with FL SDC1 or the RMKKKdel mutant expressed higher levels of SDC1 mRNA compared to the control cells, as expected (Figure 5A,B). Flow cytometry analysis demonstrated increased cell surface levels of SDC1 in B6FS cells stably overexpressing FL SDC1 or the RMKKKdel mutant compared to the control cells (Figure 5C). Brightfield microscopy revealed an elongated morphology of the control B6FS cells, which was not clearly affected by overexpressing FL SDC1 or the RMKKKdel mutant (Figure 5D, upper panels). Immunofluorescence staining showed that in contrast to A549 cells, only weak staining was detected in the cell nuclei of the control B6FS cells (Figure 5D). Nuclear staining of SDC1 was significantly increased in B6FS cells overexpressing FL SDC1, but not the RMKKKdel mutant, as expected based on previous studies showing that the RMKKKdel mutant had a deficiency in translocating to the nucleus [28].

**Figure 6:**
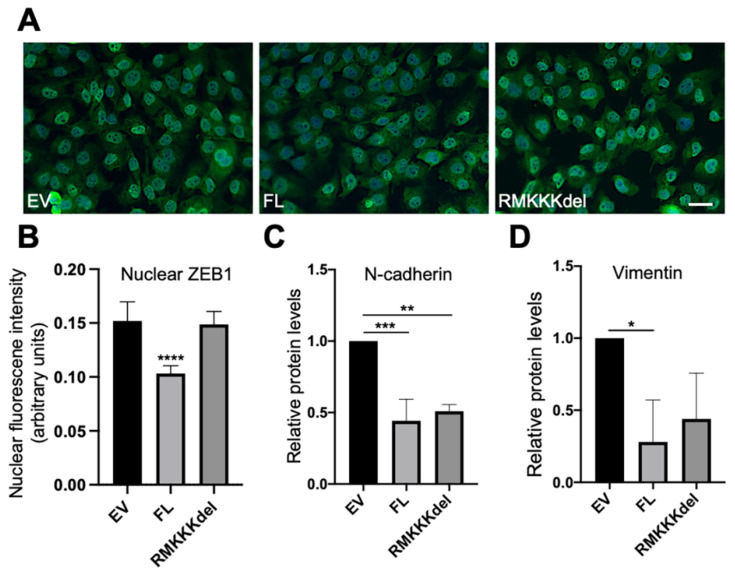
Effect of overexpression of SDC1 on EMT markers in B6FS fibrosarcoma cells. (**A**) Immunofluorescent staining of ZEB1 in B6FS cells expressing empty vector (EV), FL SDC1 (FL), or the RMKKKdel mutant. (**B**) Bar graph showing results from quantification of nuclear ZEB1 intensity in B6FS cells transfected with an EV, FL SDC1, or the RMKKKdel mutant. (**C**,**D**) Bar graphs showing protein levels of N-cadherin and vimentin in B6FS cells transfected with an EV, FL SDC1, or the RMKKKdel mutant. The results represent mean values normalized to GAPDH levels and are based on three experiments. Scale bar = 10 μm. * = *p* < 0.05; ** = *p* < 0.01; *** = *p* < 0.001; **** = *p* < 0.0001.

Based on the results from the A549 cells, we analyzed whether overexpression of SDC1 would affect EMT markers in B6FS cells. Immunofluorescent analysis revealed intense nuclear staining of ZEB1 in the control B6FS cells (Figure 6A). In comparison, reduced nuclear ZEB1 intensity was observed in BSFS cells overexpressing FL SDC1, but not in cells overexpressing the RMKKKdel mutant (Figure 6A,B). Western blot analysis showed that E-cadherin levels were undetectable in control B6FS cells, and this did not change after overexpression of FL SDC1 or the RMKKKdel mutant (Supplementary Figure S3A). In contrast, N-cadherin levels were significantly reduced in B6FS cells overexpressing either the FL SDC1 or the RMKKKdel mutant compared to the control cells (Figure 6C and Supplementary Figure S3B). Vimentin levels were significantly reduced in B6FS cells overexpressing FL SDC1, and showed a tendency to also be repressed in B6FS cells overexpressing the RMKKKdel mutant, but this was not statistically significant (Figure 6D and Supplementary Figure S2C).

### 3.4. Nuclear SDC1 Inhibits the Invasive Properties of B6FS Fibrosarcoma Cells

**Figure 7:**
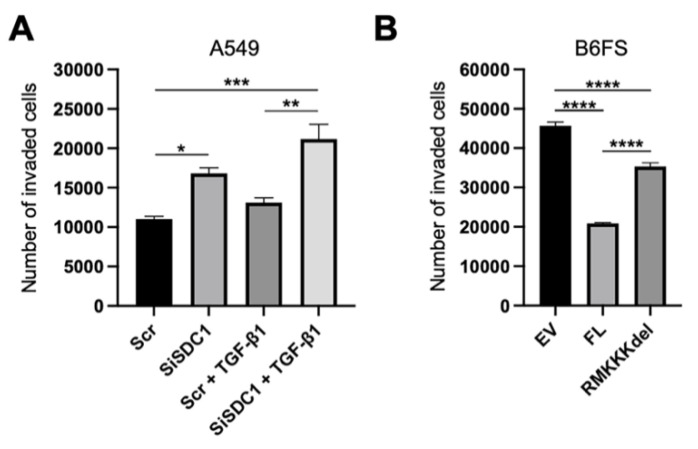
Impact of nuclear SDC1 on tumor cell invasion. (**A**) Bar graphs showing the impact of SDC1 knockdown +/−TGF-β1 treatment on the invasive capacity of A549 cells. (**B**) Bar graph showing the effect of overexpressing FL SDC1 or the RMKKKdel mutant on the capacity of B6FS cells compared to the empty vector (EV). The results represent the mean values of three independent experiments. * = *p* < 0.05; ** = *p* < 0.01; *** = *p* < 0.001; **** = *p* < 0.0001.

Together, the results from the A549 and the B6FS tumor cell models indicated that nuclear SDC1 plays a role in regulating epithelial-mesenchymal plasticity in human tumor cells. To explore the functional impact of this, we performed invasion assays with both model systems. The results showed that knockdown of SDC1 promoted increased invasion of A549 cells, both in the absence and presence of TGF-β1 (Figure 7A). Conversely, overexpression of FL SDC1 inhibited the invasive properties of B6FS cells significantly more efficiently compared to the RMKKKdel mutant (Figure 7B).

## 4. Discussion

Epithelial-mesenchymal plasticity is implicated in regulating the capacity of tumor cells to adapt to different microenvironments during the metastatic journey. However, the mechanisms of how tumor cells switch between epithelial-mesenchymal states are not fully understood. An integral part of this phenotypic plasticity is shifting the repertoire of cell surface proteins that are expressed in tumor cells. SDC1, the major cell surface proteoglycan in epithelial cells is deregulated during the EMT process. Based on recent data showing that SDC1 can translocate to the cell nucleus in tumor cells, we set out to study whether nuclear SDC1 might play a role in regulating epithelial-mesenchymal plasticity.

The results revealed loss of nuclear SDC1 during TGF-β1-induced EMT in A549 cells. Further studies showed that siRNA-mediated silencing of SDC1 enhanced the EMT response to TGF-β1 as evident by an accentuated E-to-N-cadherin switch and increased nuclear localization of ZEB1. Overexpression of full length SDC1 was significantly more effective than the RMKKKdel mutant of SDC1, which lacks the nuclear translocation signal, in repressing mesenchymal features of B6FS fibrosarcoma cells. In particular, overexpression of the RMKKKdel mutant did not repress nuclear ZEB1 expression and inhibit the invasive capacity of B6FS cells to the same extent as full length SDC1.

Consistent with our results, others have reported that SDC1 regulates EMT in tumor cells. Knockdown of SDC1 promoted EMT, while overexpression of SDC1 induced epithelial characteristics in mammary tumor cells and oral cancer cells [15,29]. Interestingly, ZEB1 was recently identified as a repressor of SDC1 in prostate cancer cell lines displaying EMT characteristics [15]. Together with our results, this indicates the possible existence of a feedback loop through which SDC1 and ZEB1 inhibit the activity of each other. The results showing that knockdown of SDC1 resulted in reduced E-cadherin levels are in line with recent studies in mesothelioma cells [16], and may indicate that SDC1 plays a role in regulating the activity of several core EMT-TFs.

Based on our results, it will be interesting to elucidate the more specific role of nuclear SDC1 for the regulation of EMT in tumor cells. It is possible that different mechanisms apply to different tumor cells based on their origin and which signaling pathways and downstream mediators drive the EMT program. On this topic, ZEB1 was identified as a transcriptional repressor of SDC1 in prostate cancer cells [17]. In contrast, positive correlation between Snail expression and nuclear translocation of SDC1 has been reported in prostate cancer cells [30]. These somewhat conflicting data indicate that the interactome of nuclear SDC1 might differ in different tumor cells. Further studies are needed to elucidate the mechanisms of how nuclear SDC1 regulates different components of the EMT machinery.

Intriguingly, we recently found that SDC1 interacts with a large number of nuclear proteins in human mesothelioma cells including proteins involved in RNA splicing and ribosomal biogenesis [31]. Based on reports showing that in addition to transcriptional reprogramming, alternative splicing and increased ribosomal biogenesis contribute to an expansion of the cell proteome during EMT [32,33], it will be interesting to investigate whether nuclear SDC1 regulates epithelial-mesenchymal plasticity by interfering with these post-transcriptional processes. Related to this, it was recently found that Snail regulates mRNA/miRNA interactions in colorectal cancer cells [34].

In conclusion, we demonstrated that nuclear translocation contributes to the capacity of SDC1 to regulate EMT characteristics and the invasive behavior of tumor cells. The results provide new insight into the mechanisms by which SDC1 plays a role in EMT and tumor progression and open up further studies to explore the mechanisms by which nuclear SDC1 regulates the EMT machinery in tumor cells.

## Data Availability

Not applicable.

## Supplementary Materials

The following are available online at https://www.mdpi.com/article/10.3390/biology10060521/s1, Figure S1: SDC1 mRNA expression in human cancer cells of different origin, Figure S2: Effect of SDC1 knockdown on EMT markers in A549 cells, Figure S3: Effect of SDC1 overexpression on EMT markers in B6FS fibrosarcoma cells.
