# Supplementary material for: Nuclear Syndecan-1 Regulates Epithelial-Mesenchymal Plasticity in Tumor Cells
Source: Biology (Basel). 2021 Jun 11;10(6):521. doi: 10.3390/biology10060521 (PMC8230654; doi:10.3390/biology10060521)
Supplement: Supplementary file 1 [file biology-10-00521-s001.zip › biology-1196512-supplementary.pdf]

biology

MDPI

Supplementary Materials

Nuclear syndecan-1 regulates epithelial-mesenchymal plasticity in tumor cells

Ashish Kumar-Singh, Malgorzata Maria Parniewska, Nikolina Giotopoulou, Joman Javadi, Wenwen Sun, Tünde Szatmári, Katalin Dobra Anders Hjerpe and Jonas Fuxe

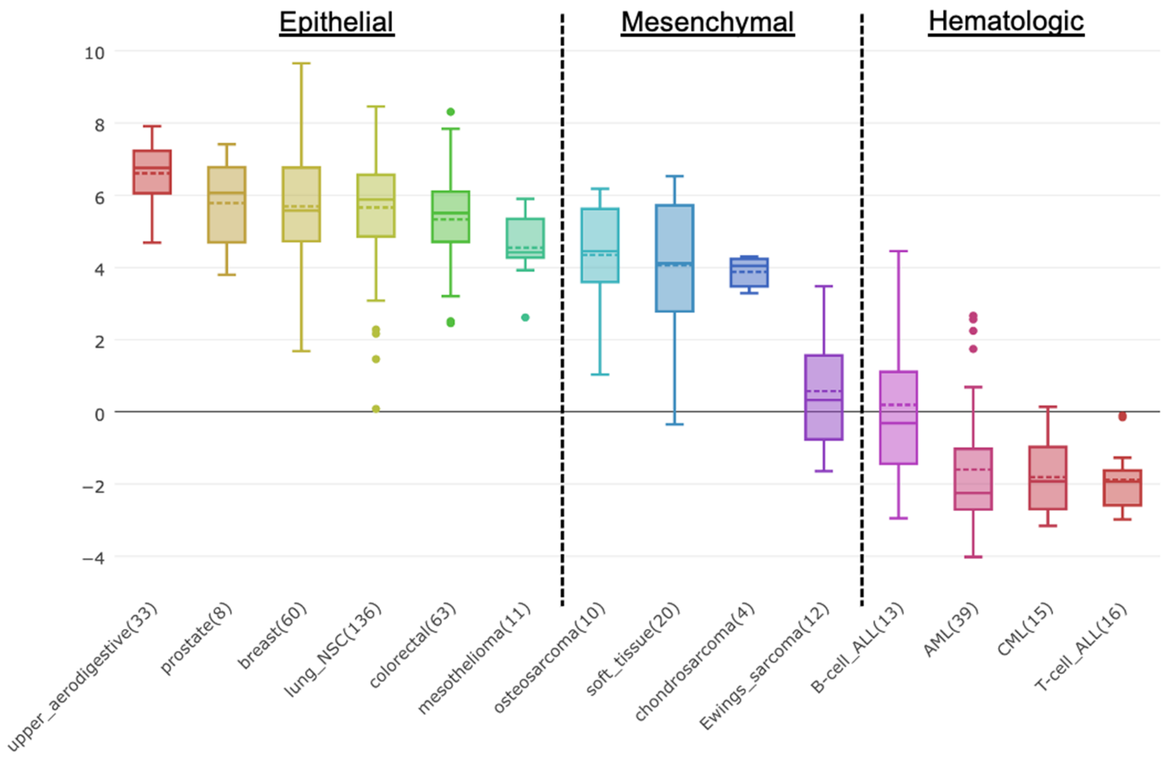

Figure S1. ***SDC1*** **mRNA expression in human cancer cells of different origin.** Graph showing relative *SDC1* mRNA expression in human cancer cells with epithelial, mesenchymal and hematologic origin. Values within parenthesis indicate the number of analyzed cell lines per tumor type. The RNA sequencing data were obtained from the Broad Institute Cancer Cell Line Encyclopedia (https://portals.broadinstitute.org/ccle).

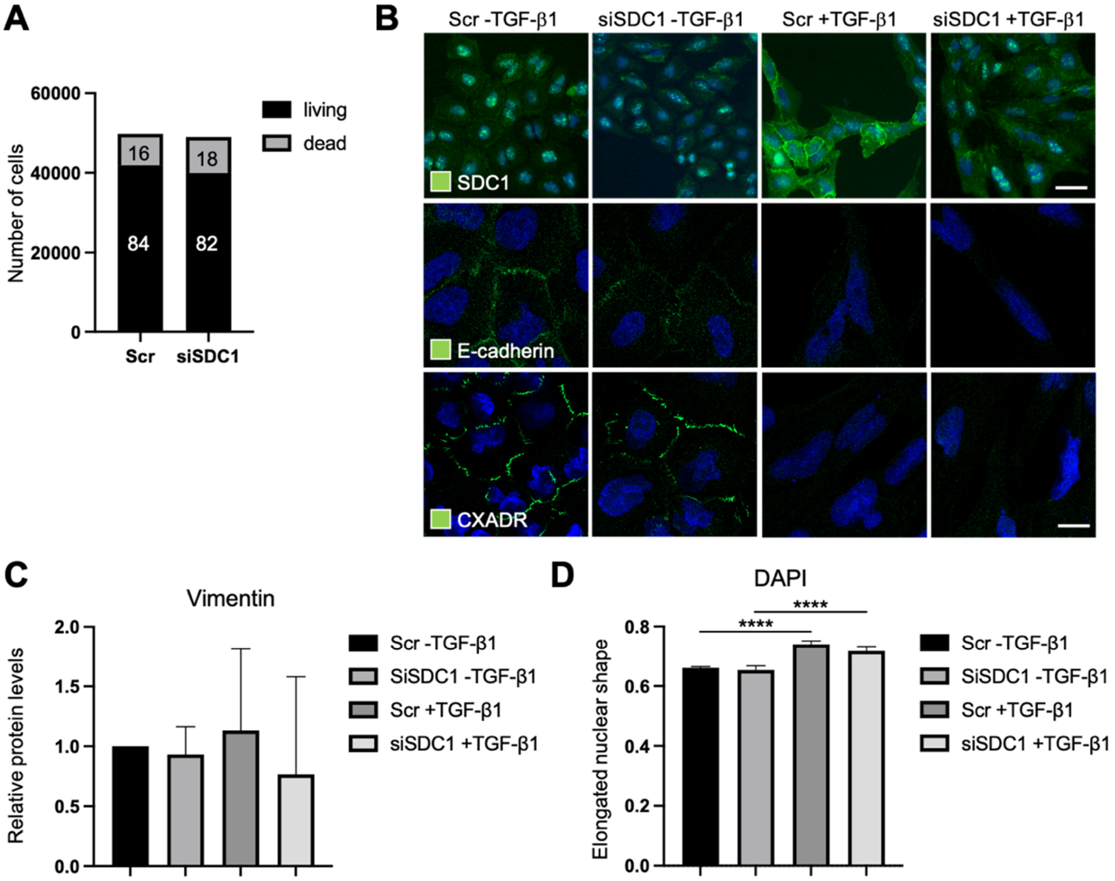

Figure S2. Effect of SDC1 knockdown on EMT markers in A549 cells. (A) Bar graph showing minor toxic effects in A549 cells after transfection of either scrambled control siRNA (Scr) or SDC1 siRNA (siSDC1). The numbers in the bars indicate the percentage of living and dead cells in each of the samples. (**B**) Immunofluorescence images showing staining of SDC1 and the epithelial markers E-cadherin and CXADR in A549 cells transfected with scrambled control siRNA (Scr) or SDC1 siRNA (siSDC1), and thereafter left untreated or treated with TGF-β1 (10ng/ml, 72 h). Cell nuclei were counterstained with DAPI (blue). Scale bars = 10 µm (upper panels);.5 µm (middle and lower panels). **(C)** Bar graph showing quantification of western blot experiments studying the effect of SDC1 knockdown and TGF-β1 treatment (10ng/ml, 72 h) on vimentin protein levels. (**D**) Bar graph showing the effect of SDC1 knockdown and TGF-β1 treatment (10ng/ml, 72 h) on elongated nuclear shape.

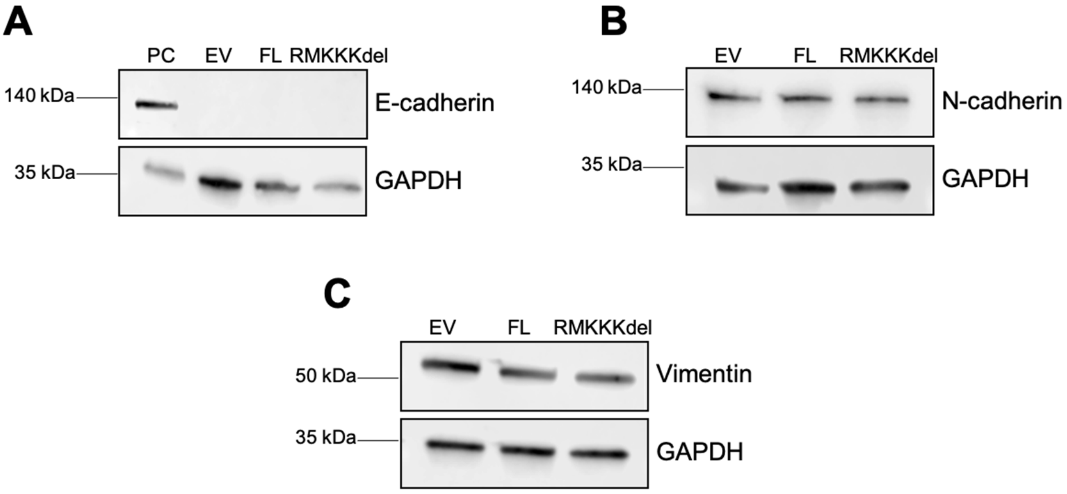

Figure S3. Effect of SDC1 overexpression on EMT markers in B6FS fibrosarcoma cells. (A-C) Western blot results showing the effect of stable overexpression of an empty vector (EV), full length (FL) or a mutated SDC1 lacking the nuclear targeting sequence (RMKKKdel) on the protein levels of E-cadherin (**A**), N-cadherin (**B**) and vimentin (**C**). Cell lysates from the human PC3 prostate cancer cell line (PC) was used as a positive control for E-cadherin. GAPDH was used as a loading control.
